# First Report of an Invasive Infection by *Cephalotrichum gorgonifer* in a Neutropenic Patient with Hematological Malignancy under Chemotherapy

**DOI:** 10.3390/jof7121089

**Published:** 2021-12-17

**Authors:** Ana Álvarez-Uría, Pilar Escribano, Verónica Parra-Blanco, José Francisco Cano-Lira, Alberto Miguel Stchigel, Gillen Oarbeascoa, Patricia Muñoz, Jesús Guinea

**Affiliations:** 1 Clinical Microbiology and Infectious Diseases Department, Hospital General Universitario Gregorio Marañón, Universidad Complutense de Madrid, 28007 Madrid, Spain; ana_miyares@yahoo.es (A.Á.-U.); pmunoz@hggm.es (P.M.); 2 Instituto de Investigación Sanitaria Hospital Gregorio Marañón, 28007 Madrid, Spain; gillen.oarbeascoa@salud.madrid.org; 3 Pahological Anatomy Department, Hospital General Universitario Gregorio Marañón, Universidad Complutense de Madrid, 28007 Madrid, Spain; vero@rpiedra.com; 4 Unitat de Micologia, Facultat de Medicina i Ciències de la Salut, Universitat Rovira i Virgili, 43204 Reus, Spain; jose.cano@urv.cat (J.F.C.-L.); albertomiguel.stchigel@urv.cat (A.M.S.); 5 Hematology Department, Hospital General Universitario Gregorio Marañón, Universidad Complutense de Madrid, 28007 Madrid, Spain; 6 CIBER Enfermedades Respiratorias-CIBERES (CB06/06/0058), 28029 Madrid, Spain; 7 Medicine Department, School of Medicine, Universidad Complutense de Madrid, 28007 Madrid, Spain

**Keywords:** *Cephalotrichum gorgonifer*, invasive fungal infection, neutropenia, molecular identification, diagnosis

## Abstract

The etiological agents of infrequent invasive fungal infections (IFI) are difficult to identify on the species level using classic morphological examination. We describe the first case of an IFI caused by *Cephalotrichum gorgonifer* in a neutropenic patient with a hematological malignancy and put it on the map as a new causative agent of IFI. Case report, microbiological findings and description of the etiological agent. A 60-year-old man was diagnosed with mantle cell lymphoma. A CT scan confirmed the presence of lung infiltrates located at the right upper lobe. Histological examination of one of the nodules showed a large number of narrow septate hyphae with acute-angle branching and irregular round cell morphology; vessels walls appeared infiltrated, proving an angioinvasive pulmonary IFI. Sample culture resulted positive and molecular identification proved the presence of *Cephalotrichum gorgonifer*. Voriconazole was used for 12 months and the patient did not report any complications or side effects. Complete remission of lymphoma was achieved later by the time chemotherapy, radiotherapy, and radioimmunotherapy consolidation were completed. We recommend the inclusion of *Cephalotrichum gorgonifer* in the list of opportunistic pathogens causing mycoses in neutropenic hematological patients with suspected mould-related IFI.

## 1. Introduction

*Aspergillus* spp. are the main cause of invasive fungal infections (IFIs) in patients with hematological malignancies or in hematopoietic stem cell transplant recipients [1,2]. However, changes in epidemiology and in antifungal prophylaxis strategies are leading to an increasing number of other emerging opportunistic moulds causing IFIs [2,3]. The etiological agents of infrequent IFIs are difficult to identify on the species level using classic morphological examination; therefore, molecular techniques may be helpful for the identification of such fungal pathogens [3,4].

Here, we describe the first case of an IFI caused by *Cephalotrichum gorgonifer* (Bainier) Sand.-Den., Gené & Guarro, in a neutropenic patient with a hematological malignancy and put it on the map as a new causative agent of IFIs.

## 2. Case Presentation

A 60-year-old man with a cervical mass was diagnosed with mantle cell lymphoma in January 2007; intensive chemotherapy and rituximab were initiated. After the second cycle given in February, he developed severe neutropenia and persistent fever despite the administration of broad-spectrum antibiotics. An X-ray showed peripheral lung nodules and IFI was suspected; empirical antifungal treatment with voriconazole was included. A CT scan confirmed the presence of lung infiltrates, one of which was round-shaped, 3 cm long, showed a mixed pattern, and was located at the right upper lobe; the other nodule was smaller and located at the left lower lobe.

**Figure 1:**
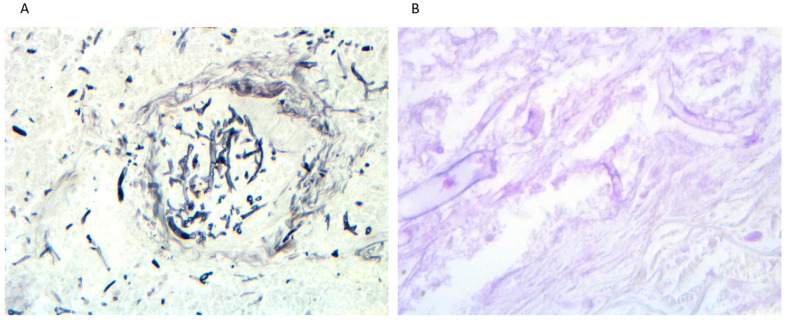
Lung tissue sections and histological examination showing the presence of septate hyphae, some causing angioinvasion. ((**A**) Grocott stain, magnification 200×) and ((**B**) PAS staining, magnification 400×).

**Figure 2:**
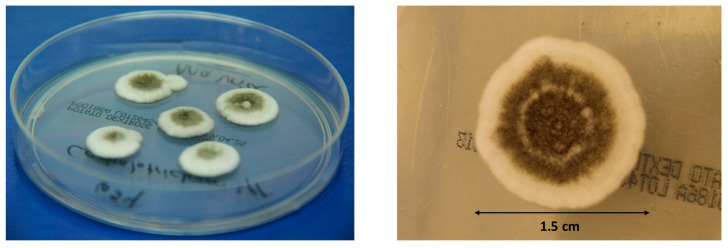
*Cephalotrichum gorgonifer* on potato dextrose agar plates after 10 days of incubation at 35 °C.

A percutaneous lung biopsy was taken and microbiological cultures of the sample remained negative; surgical resection of the larger pulmonary nodule was performed in March. Histological examination (PAS/Grocott) showed an abscessed area with necrosis containing a large number of narrow septate hyphae with acute-angle branching and irregular round cell morphology; vessels walls appeared infiltrated, proving an angioinvasive pulmonary mycosis (Figure 1). Fungal cultures of the sample yielded a hyaline fungi microscopically identified as *Scedosporium* spp. (Figure 2).

Fourteen days later, the patient recovered from neutropenia, but developed left visual field loss. A brain CT scan showed several cerebral lesions particularly in his right occipital lobe. A cerebral biopsy was performed and histological examination (PAS/Grocott) showed spongiosis of the cerebral parenchyma, hypertrophic astrocytes with eosinophilic cytoplasm, and an area of necrosis with abundant erythrocytes. No structures resembling fungi were detected at the time and fungal culture was negative. Voriconazole antifungal treatment was maintained and patient’s clinical condition improved, although the fever persisted for several days. He was finally discharged three months after admission with partial remission of the lymphoma and radiotherapy was scheduled. A CT scan was performed one month after discharge that showed enlargement (3.8 cm diameter long) of the previous nodular lesion in the right upper lung lobe, now with necrosis and satellite nodules. The patient was admitted for a right upper lobectomy. The histological examination again showed numerous fungal structures similar to those previously described, along with fibrosis and interstitial haemorrhage and a mature inflammatory infiltrate with round nucleus lymphocytes and multinucleated giant cells of foreign body type. This time, fungal cultures were negative. Another CT scan was performed two months later and showed residual fibrotic scarring tracts in both upper lobes; no new nodules, infiltrates, or pathologically enlarged adenopathies were detected.

Both paraffin-embedded tissue sections were processed for fungal DNA extraction using a paraffin-embedded specific extraction kit (QIAmp DNA FFPE tissue kit, Hilden, Germany) and the area of the sample where DNA extraction was carried out was guided by the pathologist. PCR amplification of the ITS1-ITS-2 region and *β-tubulin* performed on both paraffin-embedded tissue sections, in which fungal-resembling elements were seen, were negative.

Voriconazole was maintained for 12 months and the patient did not report any complications or side effects. Complete remission of lymphoma was achieved 12 months later by the time chemotherapy, radiotherapy, and radioimmunotherapy consolidation were completed. Several years later, the patient underwent a matched sibling allogeneic stem cell transplantation with no infectious complications. At the writing of this manuscript, the patient is alive and his hematological disease remains in complete remission.

**Figure 3:**
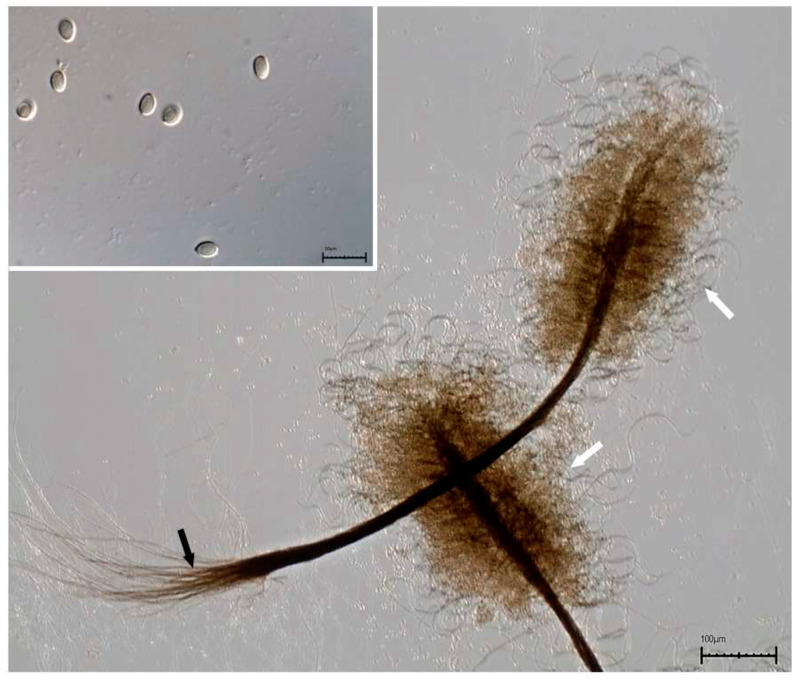
Typical morphology of *Cephalotrichum gorgonifer*. The main image shows two synnemata with long rhizoids (black arrow) and a head ornamented with curled setae (white arrow). The small image shows the conidia, which are one-celled, pale brown, smooth-/thick-walled, and ellipsoidal conidia pointed at the apex and truncated at the base.

In 2018, we studied the epidemiology of scedosporiosis affecting patients cared for at *Hospital Universitario Gregorio Marañón* from 1998 to 2017 [5]. Available *Scedosporium*/*Lomentospora* strains were retrieved for molecular identification using the ITS-1-ITS-2 region. This approach helped us correctly identify the aforementioned isolate recovered from a lung tissue sample as *C. gorgonifer*. In culture, the colonies on potato dextrose agar (PDA; Pronadisa, Madrid, Spain) at 25 °C achieved a diameter of 72–78 mm after 14 days of incubation; initially, they were hyaline, velvety to slightly cottony, becoming grey and fasciculate with age, particularly in the center part in their upper surface, and grey on reverse view. Colonies on oatmeal agar (OA; 30 g of filtered oat flakes, 15 g agar-agar, 1 L tap water) at 25 °C attained a diameter of 58–65 mm after 14 days of incubation, were much more flattened, the aerial mycelium was absent, and the mycelium (mostly submerged) was grey and fasciculate in the center. Hyphae were subhyaline to pale brown, septate, smooth-/thin-walled, and 2–4 µm wide. Conidiophores arising from the substratum were erect, unbranched, septate, smooth-walled, grouped in synnemata (Figure 3). Synnemata appeared with a nearly black stipe and rhizoids, 450–2500 μm long (including the rhizoids), 20–35 μm wide at the middle part bearing the fertile region at the tip; the fertile region formed a more or less cylindrical sporulating head of 300–700 μm long; numerous rhizoids, septate, brown, mostly unbranched, 200–950 μm long, were present; sterile setae formed at the upper part of the synnemata septates, which were branched, smooth-/thin-walled, pale brown, coiled, up to 500 μm long and 3–4 µm wide. Conidiogenous cells were found to be annellidic, subhyaline to brown, smooth-/thin-walled, flask-shaped, 7–9 × 3–4 µm, arranged in a penicillate pattern on septate lateral branches, arising from the main axis of the conidiophores. Conidia were enteroblastic, one-celled, dry, produced in basipetal chains, smooth-/thin-walled, pale brown to pale greyish brown, obovoid, 5–6 × 3–3.5 μm, slightly darker and truncate at the base and pointed at the apex.

## 3. Discussion

Here, we describe the first case of invasive disease caused by *C. gorgonifer* in humans. The *Microascaceae* family is a heterogeneous group of fungi that includes plant pathogens, saprobes, and opportunistic human pathogens, as the species of the genera *Scedosporium* and *Scopulariopsis* [6]. *Scedosporium* and *Scopulariopsis* have been more widely studied due to their clinical relevance. The genus *Cephalotrichum*, another member of the *Microascaceae* family, is characterised by the formation of dry-spored synnemata and enteroblastic percurrent conidiogenesis [7]. Some strains have been isolated from hair and the respiratory tract in humans and are considered mere colonizers [8]. *Cephalotrichum* spp. are mostly recovered from decaying plant material, wood, and soil, and sometimes from the indoor or built environments [8,9,10]. Air-borne conidia originated at such sources could have been inhaled by the patient who developed the infection here reported.

The isolate was originally misidentified as *Scedosporium* sp., but the molecular approach helped us correctly identify it as *C. gorgonifer*. It is fair to highlight the role of molecular identification to obtain veracious and accurate knowledge on fungal species that cause IFIs.

The case reported here mimics the clinical presentation of other invasive mould infections in neutropenic patients with hematological malignancies. Lung and potentially the involvement of the central nervous system, along with inaccurate morphological identification of the isolate—molecular identification of fungal isolates was unavailable at the hospital at that time—were suggestive of scedosporiosis. The patient slowly recovered without any other aftermath and was able to complete his lymphoma treatment without further complications. Early antifungal treatment with voriconazole, surgery, and recovery from neutropenia were probably key for the favourable outcome of the patient. Moreover, our patient may have been less immunosuppressed and for a shorter time than other IFI cases with worse outcomes. The patient was not under antifungal treatment before developing IFI.

There are no data on antifungal susceptibility of *C. gorgonifer* in the literature, possibly because of the inability of the isolates to grow in RPMI broth medium, as observed with our case report. Finally, a limitation of this work is that we were unable to prove the presence of *C. gorgonifer* DNA in the tissue sections to prove hypha invasion. Both histological samples were processed according to a specific paraffin-embedded kit for DNA extraction, and the selection of the area for DNA extraction was guided by the pathologist. We were unable to amplify fungal DNA from the clinical sample using the same pair of primers used to amplify the pure-cultured isolate. In light of lack of amplification of the whole ITS1-5.8S-ITS2 region, we tried to amplify separately the ITS1 and ITS2 regions, again failing in such attempt. The fact that panfungal PCRs were performed 14 years after tissue section preparations may explain the false negative results, due to DNA degradation.

In conclusion, we recommend the inclusion of *C. gorgonifer* in the list of opportunistic pathogens causing IFIs in neutropenic hematological patients with suspected mould-related IFI. Molecular identification of isolates should be carried out on clinically significant fungi causing IFIs for current and accurate insight on the epidemiology of mycoses.

## Data Availability

Data sharing is not applicable to this article.
